# Observational study of missing SOFA score data frequency in RCTs relative to ICU length of stay

**DOI:** 10.1038/s41598-024-67089-4

**Published:** 2024-07-12

**Authors:** Denise Molinnus, Michael Beulertz, Johannes Bickenbach, Gernot Marx, Carina Benstoem

**Affiliations:** Department of Intensive Care Medicine. Medical Faculty, RWTH Aachen University, Aachen, Germany

**Keywords:** Prognosis, Medical research, Randomized controlled trials

## Abstract

The Sequential Organ Failure Assessment, also known as SOFA score, was introduced to assess organ dysfunction of critical ill patients. However, understanding the impact of missing SOFA scores in randomized controlled trials and how this affect the validity and applicability of the SOFA score as a surrogate endpoint for predicting mortality has been a matter of interest. To address this, a secondary analysis of a systematic review was conducted to quantify the relationship between SOFA scores and the prediction of mortality in critically ill adults in randomized controlled trials (RCTs). The systematic review being referred to included 87 RCTs with a total of 12,064 critically ill patients. This analysis focused on missing SOFA score data in relation to the length of stay on the intensive care unit (ICU) and the methods used to handle missing data. SOFA score measurements from the included studies were categorized into three time frames: *Early* (t ≤ 4 days), *Intermediate* (t = 5–10 days) and *Late* (t > 10 days) measurement. Only one study reported a complete data set for calculating the SOFA score for an *Early* measurement. When considering all methods used to address missing data, 32% of studies still had missing data for *Early* measurements, and this percentage increased to 64% for *Late* measurements. These findings suggested that, over time, the number of studies with incomplete data sets has been increasing. The longer a patient is treated on the ICU, the higher the number of missing data which can impact the validity of SOFA score analyses. There was no clear trend towards a specific method for compensating missing data. An exemplary calculation demonstrated that ignoring missing data may lead to an underestimated variability of the treatment effect. This, in turn, could bias the interpretation of study results by policy- and clinical decision-makers. Overall, there are several limitations that need to be considered when using SOFA score as a surrogate endpoint for mortality. When employed as an outcome, the SOFA score is frequently missing and most studies do not adequately describe the amount or nature of missing data, or the methods used to handle missing data in the analysis.

## Introduction

The *Sequential Organ Failure Assessment* (SOFA) score, previously known as the *Sepsis-related Organ Failure Assessment score*, was introduced by Vincent et al.^1^ to assess organ dysfunction across six organ systems. It has been widely implemented in research to ensure comparability in clinical trials^2^. Ref^1^ emphasized that SOFA scores can help describe a sequence of complications in critically ill patients rather than predict their outcome. One significant advantage is that there is no separate evaluation of the individual function of each organ by existing severity indices^1^.

Today, however, the SOFA score is frequently used as a primary or secondary endpoint in randomized controlled trials (RCTs), often serving as a surrogate for mortality^3,4^. This approach has become common practice as it may demonstrate higher sensitivity to treatment effects than mortality itself, enabling the analysis of smaller sample sizes. More recently, de Grooth et al.^5^ conducted a meta-analysis to examine the association between the SOFA score and mortality during a trial. They evaluated 87 RCTs comparing SOFA score and mortality between treatment groups and concluded that SOFA is a reliable and consistent surrogate parameter for mortality in RCTs.

Missing data is a prevalent issue in clinical trials^6,7^, however, this does not inherently compromise validity which is depending on the associations studied. Any resulting bias depends on the missing data mechanism. This may result in overestimation or underestimation in case of bias, and underestimation in case of noise^8,9^. To describe missing data, common categories such as missing completely as random (MCAR), missing at random (MAR) and missing not at random (MNAR) are generally used^10^.

Studies involving SOFA score can also affected by missing data. The presence of missing data is a significant concern as observations lacking data on all analytic variables cannot be included into the analysis, and only observations with complete data will be selected into the final analytic sample. This selection may bias the relationship between exposure, outcome and other variables^11^. For instance, critically ill patients may differ between their severity of their illness and the complexity of their ICU treatment. Additionally, in terms of the time course and assessment of the SOFA score, some patients may have already improved and been discharged from the ICU, or in the worst case, may have already died. In both scenarios, the SOFA score can no longer be assessed. Moreover, the absence of at least one SOFA component may hinder the correct calculation of the entire score. Consequently, excluding patients with missing SOFA scores from an analysis may lead to invalid interpretation of results. On the other hand, ignoring missing data reduces statistical power, increases bias, and underestimates response variability, as stated in the European Medicines Agency (EMA) guideline for missing data of the primary endpoint in confirmatory trials^12^. Until today, there is no common approach for handling missing SOFA scores in RCTs^5,13^. Hence, there is an urgent need to investigate the relationship between SOFA score and mortality in the context of the systematic occurrence of missing SOFA scores; its impact on the validity of study results is largely unknown.

The present study investigates the significance of missing SOFA scores in RCTs of critically ill patients, explores the common methods used to handle missing SOFA scores, and assess how ignoring missing data affects the validity and applicability of SOFA score as a surrogate endpoint to predict mortality.

## Materials and methods

### Definition SOFA score

The SOFA score measures the functioning of six different organ systems known as SOFA components: the respiratory (PaO_2_/FiO_2_), cardiovascular (mean arterial pressure or administration of vasopressors required), hepatic (bilirubin), coagulation (platelets × 10^3^/mL), renal (creatinine, urine output) and neurological system (Glasgow Coma Scale). Typically, the SOFA score is calculated upon admission to the ICU and at 24 h intervals. Each SOFA component has a range of 0 to 4 points, resulting in a total score of 0 to 24 points^1^.

In some cases, the neurological system is excluded to generate a modified SOFA score that ranges from 0 to 20 points^2^. This is due to the limitation that patients in coma cannot be properly assessed using the Glasgow Coma Scale.

Various statistical outcome measures related to the SOFA score have previously been utilized, including maximum SOFA score (the highest score during a specific time period), delta SOFA (the SOFA score at a specific time point during the trial minus the baseline score), SOFA score at a specific point in time (e.g., admission to the ICU)^5,14,15^, or a combination of these measurements (e.g., delta of maximum and baseline SOFA scores).

### Data collection

The systematic review and meta-regression analysis by de Grooth et al.^5^ served as basis for this secondary analysis. They conducted a comprehensive literature search to identify all published RCTs that reported mortality as well as common SOFA derivatives as a clinical outcome. A total of 87 RCTs met the inclusion criteria, providing a sufficiently broad dataset to address the research question regarding missing SOFA scores and their handling (Supplemental Digital Content 1, Details of Included Studies). The literature search included electronic databases, namely Medline and Embase, and was limited to reports in the English language. The eligible RCTs included in the analysis focused on adult ICU patients reporting both a derivative of SOFA and a measure of mortality as primary or secondary outcome. Among other data less relevant to the present investigation, the type of SOFA score measured (i.e., regular or modified), as well as the SOFA derivative analyzed (e.g., max SOFA, delta SOFA, SOFA time course) were recorded for each eligible RCT.

#### Quantification of missing SOFA scores

The included studies reported specific days on which SOFA was assessed during the patients` stay in the ICU. To facilitate data analysis, we categorized these continuous time data into three distinct time frames:

- *Early* SOFA measurements (t ≤ 4 days),
- *Intermediate* SOFA measurements (t = 5 to 10 days),
- *Late* SOFA measurements (t > 10 days),

Here, t represents the day when patients were enrolled as study participants (usually upon admission to the ICU).

This categorization allowed us to illustrate the progression of proportions of missing SOFA score over time since only a few assessments were conducted on the exact same day during ICU stay.

These three time frames were chosen to represent common use cases of SOFA assessment. It is commonly assessed upon admission to the ICU, later during ICU stay to document disease progression^16^, and occasionally as a follow-up measure (e.g., at 28 days). Even if SOFA may have been assessed more frequently, only one SOFA measurement was included per time frame, resulting in zero to three measurements per study. Each included measurement was always the latest one conducted within the respective time frame (*Early* = day 4, *Intermediate* = day 10, *Late* = latest measurement reported).

Any data not reported by de Grooth et al.^5^ were extracted directly from the original publications. For each RCT, the amount of missing SOFA scores for each time frame was documented and categorized according to the reason for missing data. Three common reasons for missing data were identified:

1. *Reason 1*: Singular missed assessments of SOFA or its components.
2. *Reason 2*: Systematically missed assessments of SOFA due to patient discharge from the ICU or hospital.
3. *Reason 3*: Systematically missed assessments of SOFA due to patient death.

*Reason 1*—singular missed assessment of SOFA or one of its components—was quantified via descriptions of missing data in the published results of the RCTs, which were mostly available as absolute values. Missing SOFA scores due to early discharge (*Reason 2)* were estimated by assessing reports of length of stay (Q1, median and Q3, minimum ICU/hospital stay). For instance, if SOFA was assessed on day 4, while median and first quartile of ICU stay were 5 and 3 days, respectively, it could be assumed that more than 25% of patients had been discharged before this *Early* measurement. Hence, *Reason 2* missing data were only available as proportions and were categorized into five groups (0%, 1–25%, 26–50%, 51–75%, > 75%). It was generally assumed that SOFA assessments were conducted throughout the entire hospital stay unless it was specified that measurements were only taken during ICU stay. Missing data due to patients death (*Reason 3)* were quantified using the reported mortality rates available as either absolute values or proportions. Here, it was generally assumed that if SOFA score assessment and report of mortality fell on the same day (e.g., SOFA measured at day 4 and mortality reported for day 4), SOFA could still be assessed on that day, unless it was specified otherwise. In some cases, mortality rates were estimated from Kaplan–Meier survival plots.

### Description of methods used to handle missing SOFA scores

For each of the three predefined reasons for missing data, it was examined whether and how patients with missing data were handled. Following the EMA guideline, this study investigated the methods employed to address missing data, including strategies to compensate for these missing values. These strategies encompassed replacing a missing value with a previously defined fixed value, such as assigning a SOFA score of 24 for death, utilizing last observation carried forward (LOCF), excluding missing values from analysis, or applying the mean of adjacent values, excluding missing values from analysis or using the mean of adjacent values, as described by Vincent et al.^17^ or Ferreira et al.^14^. Additionally, some studies employed specific statistical methods^18^, such as multiple imputation, to address missing data comprehensively. While the latter by default addresses all missing data points without being able to differentiate between the origin or nature of missing data, the other methods were interpreted individually for each form of missing data (*Reasons 1–3*), depending on how authors of the original publications reported their handling of the various forms of missing data. Some of these methods, however, are unfit to handle each form of missing data by default, e.g., calculating the mean of adjacent values is not possible for *Reason 2* or *3* missing data, where all following values are missing.

Due to the unavailability of the missing pattern structure of the source date, it was not possible to conduct a case-by-case evaluation of whether the statistical methods employed by the studies adequately accounted for missing data. Instead, we primarily assumed that the chosen strategies were appropriate for addressing the missing data.

### Exemplary calculation of the impact of complete case analysis on *bias* and estimated variance

According to the Prentice criteria^19^ for an adequate surrogate parameter, the difference between treatment groups should encompass all essential information about the true endpoint (mortality). Therefore, the effects observed on the true clinical endpoint in the analysis set should also be applicable to the surrogate parameter. In this context, bias refers to a systematic influence of missing information on the outcome variable.The aim of this calculation was to demonstrate that using only the complete case set, compared to the full analysis set, has a significant impact on bias and the estimated variance of the true endpoint. Consequently, this effect is assumed to apply to the surrogate parameter as well. Complete case set hereby refers to data sets of subjects without any missing information while the full analysis set includes all subjects. Bias in this context means a systematic influence of missing information on the outcome variable.

Studies reporting *Intermediate* SOFA scores between 4 and 10 days and providing information about exact numbers of missing data (*Reason 1* or *3*), missed assessments, mortality rates for the specific SOFA score observation day, as well as mortality rates beyond day 10, are included. Unfortunately, for *Reason 2* missing data, no absolute values were available and these are therefore not included in this analysis. Methods addressing missing values were also not considered, as this analysis serves only as an illustration.

The absolute risk reduction (ARR) of mortality was calculated as the fraction of deaths among all subjects included in the study (full analysis set) versus deaths among subjects with available data at the specific time point (complete case set). Additionally, the respective asymptotic standard deviation (SD) is used as the treatment effect^2,20,21^. The latter was calculated following standardized approaches (e.g., outlined in Ref^20,21^). The complete case set was calculated by subtracting the number of subjects with missing data from the original sample size.

### Statistical methods

Most data in the present study are descriptive in nature. Categorical variables are summarized by counts and percentages. Continuous variables are described using medians and inter-quartile ranges (IQR) to provide a robust representation of the data spread. The only inferential statistic employed, in addition to the exemplary calculation detailed in the previous chapter, is a rank correlation analysis based to Kendall’s Tau. This analysis aims to quantify the relationship between time (in days) and the proportion of missing data (represented in five ranks where 0 = 0%, 1 = 1–25%, 2 = 26–50%, 3 = 51–75%, and 4 = 76–100% of data missing). The analyses were conducted using RStudio Software, Build 351, RStudio PBC, MA, USA.

## Results

### Description of included studies

In total, 87 studies involving 12,064 critically ill patients were analyzed. The sample size of studies varied from 15 to 1,251, with a median (IQR) of 64 (39–150).

### SOFA score assessment in included studies

SOFA score was used as the primary endpoint in 24% (21/87) of the studies. Other primary endpoints included mortality, specific laboratory markers, occurrence of complications, or treatment-free days. *Early* SOFA measurements (day 1–4) were conducted in 92% (76/87) of the studies, *Intermediate* SOFA measurements (day 5–10) in 67% (58/87), and *Late* SOFA measurements (day 11 or later) in 36% (31/87) of the studies. Seven studies assessed SOFA throughout the entire ICU duration. Additionally, ten studies assessed a modified SOFA score, which excluded the neurological component. Frequently, studies explored multiple SOFA derivatives and various combinations, with the most common approach being measuring the SOFA score at a fixed time (74/87), followed by delta SOFA (30/87), maximum SOFA (14/87), SOFA time course (11/87), and sum of SOFA scores (2/87). Detailed descriptions of individual studies can be found in Supplemental Digital Content 1.

### Quantification of missing SOFA scores

Among the 87 studies included in this investigation, only 1 explicitly reported a complete data set of SOFA scores for the *Early* measurement. However, considering the use of compensatory strategies, 8 studies had complete data sets for *Early* measurements, 7 for *Intermediate* measurements, and 5 for *Late* measurements (Table 1). Fewer studies assessed SOFA at the *Intermediate* and *Late* time points overall (Table 1, *not applicable*). Despite the absolute number of studies with incomplete data sets remains constant, the proportion of studies with incomplete data sets increased over time.

**Table 1:** Summary of studies for different time points.

	Early	Intermediate	Late
Complete^a^, no. (%)	8 (9.2)	7 (8.0)	5 (5.7)
Missing^b^, no. (%)	28 (32.2)	30 (34.5)	23 (26.4)
Unknown^c^, no. (%)	46 (52.9)	27 (31.0)	8 (9.2)
Not applicable^d^, no. (%)	5 (5.7)	23 (26.4)	51 (58.6)
Total, no. (%)	87 (100)	87 (100)	87 (100)

Frequencies of studies where data can be assumed *complete* or *missing*, where data status could not be assessed (*unknown*), or SOFA was not assessed at all at the respective time point (*not applicable*). Within the specific time frames (Early, Intermediate and Late), the SOFA score must have been recorded at least once to be considered as complete dataset.

^a^Number of studies with complete data sets assuming use of compensatory strategies yields complete data sets.

^b^Number of studies with missing data.

^c^Number of studies for which completeness of data could not be assessed.

^d^Number of studies which did not assess SOFA at the respective time point at all.

**Figure 1:**
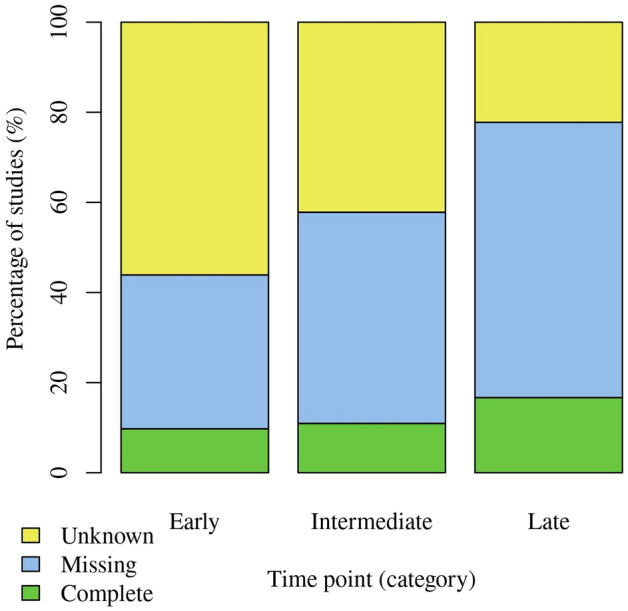
Quantity of studies with in-/complete data or unclear data status: Complete hereby means that a full set of data was explicitly mentioned, no grounds for assuming missing of data (e.g., early discharge or mortality) found, or a satisfactory effort was taken to account for missing data (e.g., use of statistical or other methods of compensation). Missing therewith refers to studies where this was not the case and unknown refers to studies where it is unclear if data was missing (i.e., lacking description of data status, no mention of satisfactory efforts of compensation nor explicit grounds for assuming the absence of data). This graph excludes irrelevant data points (i.e., studies where SOFA was not assessed at the respective time points, *not applicable*).

**Figure 2:**
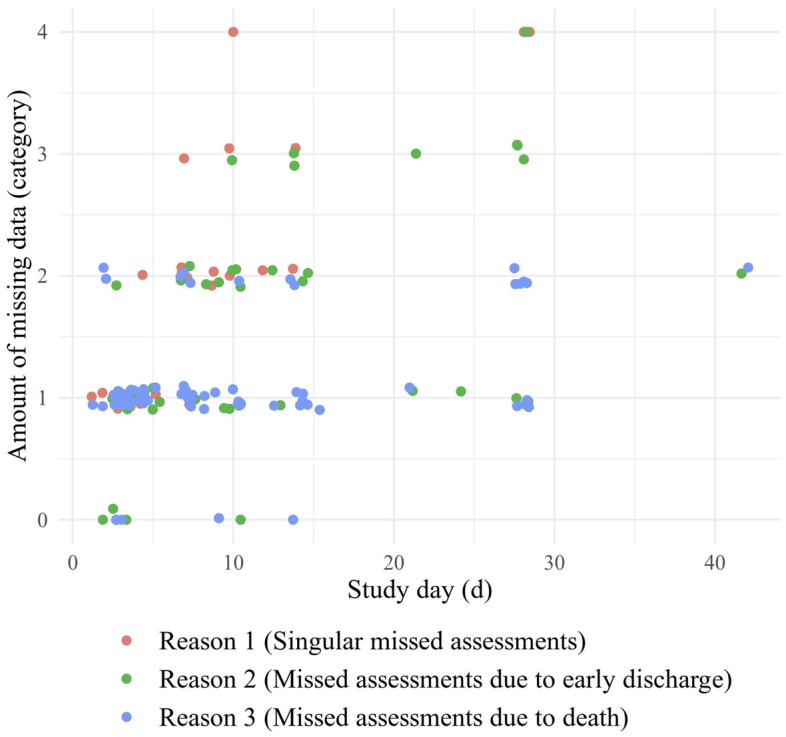
Relationship between amount of missing data within included studies and progression of time: Each dot represents a data point (i.e., data status at each time point for all studies), divided into the three reasons mentioned above. For later measurements, fewer studies show complete or almost complete data when compared to earlier measurements. 0 = 0% missing data, 1 = 1–25% missing data, 2 = 26–50% missing data, 3 = 51–75% missing data, 4 = 76–100% missing data. This graph only includes data points of category *missing* (SOFA data sets were clearly incomplete).

As depicted in Fig. 1, the number of studies lacking data increased over time, reaching 34% for *Early* measurements and 64% for *Late* measurements. To better illustrate this progression proportionally, Fig. 1 specifically excludes entries with data status *not applicable*. The number of studies with missing or unknown data status accounts for more than 80% of all studies at all times points. Figure 2 displays the course of all individual data points (up to three per study, i.e., one per time point, if applicable), indicating the total amounts of missing data for all 87 studies over time. In order to show the progression of total amounts of missing data over time, Fig. 2 only includes the data points with status *missing*. This demonstrates that there is a tendency of lower amounts of missing data (mostly 1–25%) within the first ten days and complete data sets are only found in this period. This trend disappears over time, and eventually, an equal number of studies suffer from 51 to 75% or even > 75% of missing data.

To objectify this relationship, a correlation analysis was conducted, demonstrating that the progression of time correlated significantly with missing data for all three reasons overall (Kendall’s rank correlation tau = 0.40, p < 0.001) and individually for each reason (*Reason 1*: Kendall’s rank correlation tau = 0.74, p < 0.001; *Reason 2*: Kendall’s rank correlation tau = 0.48, p < 0.001; *Reason 3*: Kendall’s rank correlation tau = 0.24, p < 0.01).

### Methods of handling missing SOFA scores

Only 3.4% (3/87) of studies considered all three reasons for missing data in terms of applying methods to compensate them. Out of the 21 studies that utilized the SOFA score as the primary endpoint, only two (10%) addressed all three reasons, comprehensively. In several studies (*Early* = 8.5%, *Intermediate* = 14.1%, *Late* = 13.9%), the authors did not specifically describe their approach to handle missing SOFA scores, even though the number of subjects in the analysis was reduced compared to the number of randomized subjects. Hence, unless within context of a complete-case analysis, this was consequently also interpreted as *Reason 1* missing data. As outlined in Table 2, missing SOFA scores due to early discharge were addressed most frequently (*Reason 2:* 15%, 13/87), followed by singular missed assessments (*Reason 1:* 13%, 11/87) and missing SOFA scores due to death (*Reason 3:* 10%, 9/87). Five studies implemented statistical methods (e.g., multiple imputations) to compensate for missing data.

**Table 2:** Methods used to consider patients with missing SOFA scores in the analysis.

Strategies	Mean of adjacent values	Fixed value	LOCF^1^	Excluded	Total
Reason 1	7	0	3	1	11
Reason 2	NA^2^	4	6	3	13
Reason 3	NA^2^	3	3	3	9
Total	7	7	12	7	33

Summary of absolute number of applied methods handling missing SOFA scores of all studies where multiple answers were possible. Please note that numbers do not sum up to 87 as not all studies applied said methods. Statistical methods are global in nature and cannot be subdivided into the aforementioned reasons for missing data, but were employed in five studies in total. *Note. Reason 1* refers to singular missed assessments, *Reason 2* refers to data missing due to discharge, and *Reason 3* refers to data missing due to death. Mean of adjacent values cannot be applied to data missing due to discharge or death (*Reason 2* and *3*), as a value before and after the missing value is required.

^1^*LOCF* last observation carried forward.

^2^*NA* not applicable.

This indicates that missing data is rarely addressed, particularly when considering that some overlap exists within these numbers: Some studies addressed multiple reasons for missing data via use of the same method and therefore attribute to more than one count in Table 2. Overall, no trend towards a specific method could be identified.

### Exemplary calculation of the impact of complete case analysis on *bias* and estimated variance

Bias and estimated variance were calculated based on data from eight studies that provided the exact (absolute) number of *Reason 1* or *3* missing data per treatment arm at the respective observation time points, along with mortality information beyond day 10. Comparing the use of the full analysis set to the complete case set revealed distinct differences: Deviation from the originally estimated treatment effect (absolute risk reduction) ranged from − 4.2 to 4.8% and almost all studies suffered from an underestimated variability of the estimated treatment effect in the range of 0.3 to 2.9%. One exception to this trend was observed in one study with the lowest mortality rates (0 overall) and smallest sample size (23 per arm). These findings demonstrate the importance of considering missing data in the analysis highlight the potential consequences of simply omitting such data in the interpretation of study results. These results are not exclusive to cases of missing data, but they serve to illustrate the broader implications of handling missing data in clinical research.

## Discussion

The screening of 87 RCTs revealed that only one study explicitly reported a complete data set of SOFA scores for the *Early* measurement. When considering the use of compensatory strategies, eight studies had complete data sets for *Early* measurements, seven for *Intermediate* measurements, and five *Late* measurement. However, the longer patients are treated in the ICU, the more likely it is for data to be missing. Furthermore, it was evident that missing data is rarely addressed in the corresponding trials.

These findings have important implications for clinicians interpreting the SOFA score as a surrogate endpoint. As patients are observed for longer periods in the ICU, the number of studies with complete data sets diminishes, affecting the accuracy of anticipated outcomes. Complete data sets become particularly valuable at this stage, as they allow for correct calculations of the SOFA score, aiding clinicians in their decision-making. Therefore, a greater effort should be made to ensure complete data collection.

The analysis also revealed that *Reason 1* missing data appears to also be related to at least one other variable, namely time, and, therefore, should be considered as missing at random (MAR) rather than missing completely at random (MCAR). Addressing missing data, weather MAR or not missing at random (MNAR) (*Reason 2* & *Reason 3*), should be prioritized to avoid potential biases in study results.

The exemplary calculation demonstrated that missing data can significantly impact the validity of SOFA score analyses, which is crucial as it serves as an essential surrogate parameter for many clinical trials. To illustrate further: an ARR of 5% translates to a number-needed-to-treat of 20, therewith every 20th person would benefit (and in the case of SOFA as a surrogate for mortality, avoiding a fatal outcome) from the intervention at hand, which can be considered as significant improvement. If the real estimate (i.e., when addressing missing data) is 5% lower, the difference would be non-existent and the same argument can be made vice versa. Aggravating this degree of uncertainty, an underestimated standard deviation of up to 3% ARR in addition to the unavoidable random error variance dilutes any precise estimate.

The implications of such missing data are substantial and can affect the interpretation of study outcomes significantly. Ignoring this issue may lead to inaccurate conclusions and can dilute the precision of estimates due to underestimated standard deviations and random error variance. Currently, no adequate methods for handling missing data in SOFA score analyses have been thoroughly evaluated. Some authors have recommended replacing single missing SOFA components (e.g., bilirubin) by calculating the mean of adjacent values^14,18^. Others have suggested using last observation carried forward LOCF^22,23^, replacement by lower value of adjacent values or setting missing values to zero if adjacent values were also missing^24^. As pointed out by the Cochrane Handbook^25^ and the EMA guidelines for confirmatory trials^17^, such fixed imputation methods (including LOCF) are generally discouraged. Excluding missing data and thereby reducing sample size via complete case analysis is only valid in case of MCAR data, whereas multiple imputation or maximum likelihood methods assume data that is at least MAR^6^, but both assumptions are clearly violated in the case of death or early discharge due to recovery. A rank-based analysis as proposed by Lachin^26^ would make it possible to include the deceased (given worst possible rank) and people who have recovered (given best possible rank) leaving only non-informative administratively missing data which could be handled through methods such as multiple imputation. General estimating equations approach can be a useful method for the analysis of longitudinal data as well as application of linear mixed models^27,28^. Both could be valuable techniques to overcome missing data in SOFA score analyses.

Additionally, different approaches to combine SOFA score and mortality measures exist. Two competing risk models were proposed by Deslandes and Chevret in 2010^29^ and Musoro et al. in 2018^16^. Toma et al.^30^ proposed an algorithm including SOFA score information to improve the prediction of survival at the end of hospital stay. Their algorithm also incorporates early discharge and missed assessment while alive on ICU. However, their approach is complex and was developed to improve an existing prediction model for mortality used by hospital administration. Further research is needed to investigate and/or develop appropriate methods to address all types of missing SOFA scores. In the meantime, other surrogate outcomes may be more suitable for studies where large amounts of missing data are to be expected.

The number of patients included in the SOFA score analysis depends in part also on the choice of SOFA derivative. Maximum SOFA score can mean that all patients with at least one SOFA score can be included in the analysis. Recent data indicates that maximum SOFA might not serve as an adequate endpoint since there is no significant correlation reported yet between maximum SOFA and mortality due to a lack of trials^5^.

This presented study has several limitations. The authors of original publications were not contacted to obtain missing information. Discrepancies within trials and information related to missing data of SOFA score were noted in Supplemental Digital Content 1. Further research is warranted to address these limitations and enhance the understanding of missing data in SOFA score analyses.

## Conclusion

This study conducted an evaluated of 87 RCTs to examine the impact of missing data on the calculation of the SOFA score and its ability to predict treatment effects on mortality. When using the SOFA score as a surrogate endpoint for mortality, several important limitations must be taken into consideration. One significant issue is the prevalence of missing data, which is often not adequately described by researchers in terms of quantity or nature, nor do they specify the methods used to handle these missing values. As time progresses, the number of missing data points tends to increase. This missing data can lead to misleading interpretations of study results and, in turn, may result in an underestimation of the variability in the estimated treatment effect. For clinical decision makers, this can have serious consequences, leading to inaccurate predictions of mortality. Therefore, it is crucial to exercise caution when using the SOFA score as a surrogate endpoint in clinical research. Understanding and properly addressing missing data is essential to ensure the validity and reliability of study findings.

### Supplementary Information

Supplementary Information.

## Data Availability

The dataset for the presented study is available from the corresponding author on reasonable questions.
