# Supplementary material for: Observational study of missing SOFA score data frequency in RCTs relative to ICU length of stay
Source: Sci Rep. 2024 Jul 12;14:16160. doi: 10.1038/s41598-024-67089-4 (PMC11245541; doi:10.1038/s41598-024-67089-4)
Supplement: Supplementary file 1 — Supplementary Information. [file 41598_2024_67089_MOESM1_ESM.pdf]

PubMed ID	author	year_published	nr	SOFA derivative_1	SOFA derivative_2	SOFA derivative_3	primary endpoint	statistical method_1	statistical method_2	statistical method_3	statistical method_IV	statistical method_compensation	reas1_mean_adjacent	reas1_LOCF	reas1_fixed	reas1_excluded	reas2_fixed	reas2_LOCF	reas2_excluded	reas3_fixed	reas3_LOCF	reas3_excluded
25862039	Payen	2015	1	4	2	1	0	3	10	888888	1	0	0	0	0	0	0	0	1	0	0	1
20495781	Acosta-Escribano	2010	2	3	1	999999	0	2	10	888888	0	0	0	0	0	0	0	0	0	0	0	0
21415104	Andrews	2011	3	4	3	888888	0	2	999999	999999	0	0	0	0	0	0	0	0	0	0	0	0
25001475	Annborn	2014	4	4	1	999999	0	4	2	10	0	0	0	0	0	0	1	0	0	1	0	0
12884156	Darenberg	2003	5	3	4	2	0	2	999999	999999	0	0	0	0	0	0	0	0	0	0	0	0
21847649	Moreno	2012	6	3	1	7	0	3	4	999999	0	1	0	0	0	0	0	0	0	0	0	0
21106341	Morrison	2011	7	3	4	999999	0	5	999999	999999	0	0	0	0	0	0	0	0	0	0	0	0
25776532	Mouncey	2015	8	3	4	1	0	6	12	999999	0	0	0	1	0	0	0	0	1	0	0	1
24108510	Papazian	2013	9	4	1	999999	0	2	6	888888	1	0	0	0	0	1	0	0	0	0	0	0
22738085	Perner	2012	10	4	1	999999	0	1	6	999999	1	1	0	0	0	0	1	0	0	1	0	0
25605469	Zhang	2015	11	5	4	999999	0	2	6	12	1	0	0	0	0	0	0	0	0	0	0	0
26444879	Lemiale	2015	12	1	4	999999	0	2	999999	999999	0	0	0	0	0	0	0	0	0	0	0	0
25241089	Sehgal	2014	13	5	4	2	0	2	8	999999	0	0	0	0	0	0	0	0	0	0	0	0
16878192	Cicarelli	2006	14	1	888888	999999	0	2	888888	999999	0	0	0	0	0	0	0	0	0	0	0	0
17174015	Mishra	2006	15	1	4	2	0	2	14	999999	0	0	0	0	0	0	0	0	0	0	0	0
14999442	Rijnders	2004	16	1	3	999999	0	10	2	888888	0	0	0	0	0	0	1	1	0	0	0	0
12163783	Caliezi	2002	17	1	4	999999	0	2	14	999999	0	0	0	0	0	0	0	0	0	0	0	0
23379498	Chu	2013	18	1	3	999999	0	9	10	999999	0	0	0	0	0	0	0	0	0	0	0	0
17992396	Cicarelli	2007	19	1	4	999999	0	888888	3	2	0	0	0	0	0	0	0	0	0	0	0	0
23351964	Eslami	2012	20	3	1	2	1	10	888888	999999	0	0	0	0	0	0	0	0	0	0	0	0
99999996	Hosny	2013	21	1	3	5	0	10	999999	999999	0	0	0	0	0	0	0	0	0	0	0	0
21118369	Huang	2010	22	1	999999	999999	0	2	999999	999999	0	0	0	0	0	0	0	0	0	0	0	0
17019548	Lauzier	2006	23	1	7	999999	0	3	4	15	0	1	0	0	0	0	0	0	0	0	0	0
22615589	Mahmoodpoor	2010	24	1	3	999999	1	1	2	999999	0	0	0	0	0	0	0	0	0	0	0	0
20080034	Manzanares	2010	25	1	2	4	0	2	999999	999999	0	0	0	0	0	0	0	0	0	0	0	0
24699555	Petros	2016	26	1	3	888888	0	10	999999	999999	0	0	0	0	0	0	0	0	0	0	0	0
23511280	Tokarik	2014	27	1	3	888888	1	10	999999	999999	0	0	0	0	0	0	0	0	0	0	0	0
12225613	Tugrul	2002	28	3	1	999999	0	1	999999	999999	0	0	0	0	0	0	0	0	0	0	0	0
21347869	Valenta	2011	29	4	1	999999	0	14	2	888888	0	0	0	0	0	0	0	0	0	0	0	0
25680826	Li	2015	30	1	3	999999	0	7	15	999999	0	0	0	0	0	0	0	0	0	0	0	0
25027749	Najafi	2014	31	1	3	999999	0	10	15	999999	0	0	0	0	0	0	0	0	0	0	0	0
21939972	Bein	2011	32	1	3	4	0	9	999999	999999	0	0	0	0	0	0	0	0	0	0	0	0
24484547	Fowler	2014	33	1	2	3	0	4	10	999999	0	0	0	0	0	0	0	0	0	0	0	0
18687132	Berger	2008	34	1	3	4	0	3	7	15	0	0	0	0	0	0	0	0	0	0	0	0
23297395	Blackwood	2013	35	1	2	3	0	10	8	888888	1	0	0	0	0	0	0	0	0	0	0	0
10321661	Briegel	1999	36	1	3	7	0	3	15	888888	0	0	0	0	0	0	0	0	0	0	0	0
24740134	Bulger	2014	37	1	3	7	0	3	6	15	0	0	0	0	0	0	0	1	0	0	1	0
24632673	Cornet	2014	38	1	5	3	0	2	999999	999999	0	0	0	0	0	0	0	0	0	0	0	0
19531784	Cruz	2009	39	1	2	3	0	10	999999	999999	1	0	0	0	0	0	0	0	0	0	0	0
20813485	Dubin	2010	40	1	3	999999	0	10	999999	999999	0	0	0	0	0	0	0	0	0	0	0	0
24342495	Gando	2013	41	1	3	999999	0	2	888888	999999	0	0	0	0	0	0	0	0	0	0	0	0
22624531	Guidet	2012	42	1	3	6	0	10	888888	999999	0	0	0	0	0	0	0	0	0	0	0	0
18504551	Iapichino	2008	43	1	3	5	0	3	14	10	1	0	0	0	0	0	0	0	0	0	0	0
17205018	Komisarof	2007	44	1	3	7	0	3	888888	999999	1	0	0	1	0	0	0	0	0	0	0	0
23348980	Kruger	2013	45	1	3	4	0	10	14	888888	1	0	0	0	0	0	0	0	0	0	0	0
26325537	Larsen	2015	46	1	4	888888	0	1	2	999999	0	0	0	0	0	0	0	0	0	0	0	0
20705780	Lasocki	2010	47	1	3	4	0	3	15	888888	0	0	1	0	0	0	0	0	0	0	0	0
25091790	Leone	2014	48	2	3	4	0	2	888888	999999	0	0	0	0	0	0	0	0	1	0	0	1
21445641	Manzanares	2011	49	1	2	3	0	3	15	14	1	0	0	0	0	0	0	0	0	0	0	0
24912653	Muller	2015	50	1	4	999999	0	2	888888	999999	0	0	0	0	0	0	0	0	0	0	0	0
26553084	Najafi	2015	51	3	1	2	0	10	15	888888	1	0	0	0	0	0	0	0	0	0	0	0
16276166	Oppert	2005	52	1	4	999999	0	11	13	888888	0	0	0	0	0	0	0	0	0	0	0	0
15286541	Reinhart	2004	53	1	3	2	0	1	2	888888	1	0	0	0	0	0	0	0	0	0	0	0
19114908	Savioli	2009	54	1	3	7	0	10	6	3	1	0	0	0	0	0	0	0	0	0	0	0
23558909	Scheeren	2013	55	5	2	888888	0	10	2	888888	0	0	0	0	0	0	0	0	0	0	0	0
22136422	Tan	2011	56	1	3	999999	0	10	2	888888	0	0	0	0	0	0	0	0	0	0	0	0
23095370	Wald	2012	57	1	2	3	0	6	3	4	1	0	0	0	0	0	0	0	0	0	0	0
23939351	Walsh	2013	58	2	4	3	0	5	888888	999999	1	0	0	0	0	0	0	0	0	0	0	0
26385664	Al tmimi	2015	59	1	4	999999	0	2	999999	999999	0	0	1	0	0	0	0	0	0	0	0	0
26853286	Barzegar	2014	60	1	3	999999	0	2	10	888888	0	0	0	0	0	0	0	0	0	0	0	0
12122526	Ralph	2002	61	1	4	5	0	2	10	888888	0	0	1	0	0	0	0	0	0	0	0	0
19559792	Richir	2009	62	4	7	1	0	2	4	888888	1	0	0	0	0	0	0	0	0	0	0	0
22366046	Schortgen	2012	63	2	1	5	0	2	10	888888	1	0	0	0	0	0	0	0	0	0	0	0
23711230	Agarwal	2013	64	2	3	888888	1	2	3	888888	0	0	0	0	0	0	0	0	0	0	0	0
18007263	Beale	2008	65	1	2	7	1	4	10	15	0	0	1	0	0	0	0	0	0	0	0	0
22692171	Brunkhorst	2012	66	1	3	4	1	10	4	999999	1	0	0	0	0	0	0	0	0	0	0	0
26373705	de Azevedo	2015	67	1	4	2	1	4	10	888888	1	0	0	0	0	0	0	1	0	0	1	0
16915107	Dubois	2006	68	1	2	3	1	10	2	999999	0	0	1	0	0	0	0	1	0	0	1	0
24781725	Erb	2014	69	1	3	7	1	10	3	11	0	0	0	0	0	0	0	0	0	0	0	0
10766680	Fagon	2000	70	1	3	999999	1	10	999999	999999	0	0	0	0	0	0	0	0	0	0	0	0
21474219	Grau-Carmona	2011	71	1	2	4	1	7	3	888888	0	0	0	0	0	0	0	0	0	0	0	0
24408035	Hall	2015	72	3	2	5	1	10	6	888888	1	0	0	0	0	0	0	0	0	0	0	0
19573904	Jabre	2009	73	5	2	3	1	10	4	999999	0	0	0	0	0	0	0	0	0	0	0	0
20663207	Pettila	2010	74	2	5	3	1	10	888888	999999	0	0	0	1	0	0	0	0	0	0	0	0
26809247	Rixen	2016	75	5	1	6	1	2	999999	999999	0	0	1	0	0	0	1	1	0	1	0	0
21658010	Wernerman	2011	76	2	7	4	1	2	999999	999999	0	1	1	0	0	0	0	1	0	0	0	0
25959848	Lu	2015	77	1	3	999999	0	10	9	888888	0	0	0	0	0	0	0	0	0	0	0	0
25851818	Pavlovic	2015	78	1	3	5	1	2	10	888888	0	0	0	0	0	0	0	0	0	0	0	0
21926601	Payen	2012	79	1	4	5	1	2	4	13	1	0	0	0	0	0	0	0	0	0	0	0
23327199	Wu	2013	80	1	3	2	0	10	3	15	1	0	0	0	0	0	0	0	0	0	0	0
26015869	Shirai	2015	81	1	4	7	0	2	888888	999999	0	0	0	0	0	0	0	0	0	0	0	0
25080051	Trzeciak	2014	82	1	2	4	0	2	14	888888	1	0	0	0	0	0	0	0	0	0	0	0
25096691	van Zanten	2014	83	1	3	4	0	10	4	888888	1	1	0	0	0	0	0	0	0	0	0	0
24105455	Wang	2014	84	1	3	888888	0	1	888888	999999	0	0	0	0	0	0	0	0	0	0	0	0
25538412	Shum	2014	85	1	2	4	1	2	999999	999999	0	0	0	0	0	0	0	0	0	0	0	0
25032942	van der Voort	2015	86	1	2	3	1	1	2	4	1	0	0	0	0	0	0	0	0	0	0	0
25561281	Zu	2015	87	1	3	999999	1	9	10	999999	0	0	0	0	0	0	0	0	0	0	0	0

PubMed ID	name_g1	name_g2	n_g1	n_g2	early_day	early_all_g1	early_all_g2	early_comp_g1	early_comp_g2	early_LOS_g1	early_LOS_g2	early_LOS_all	early_death_g1	early_death_g2	early_death_all	early_death_g1_exact	early_death_g2_exact	intermediate_day	intermediate_all_g1	intermediate_all_g2	intermediate_comp_g1	intermediate_comp_g2	intermediate_LOS_g1	intermediate_LOS_g2	intermediate_LOS_all	intermediate_death_g1	intermediate_death_g2	intermediate_death_all	intermediate_death_g1_exact	intermediate_death_g2_exact
25862039	PMX-HP	control	119	113	3	12	10	888888	888888	1	1	1	1	1	1	888888	888888	7	25	34	888888	888888	1	1	1	1	1	1	13	11
20495781	TPF	GF	50	54	4	888888	888888	888888	888888	888888	888888	888888	888888	888888	888888	888888	888888	10	888888	888888	888888	888888	888888	888888	888888	1	1	1	2	2
21415104	Glutamine+Selenium	Neither	124	125	4	888888	888888	888888	888888	888888	888888	888888	888888	888888	888888	888888	888888	7	888888	888888	888888	888888	888888	888888	888888	888888	888888	888888	888888	888888
25001475	TTM33	TTM36	71	68	4	888888	888888	888888	888888	888888	888888	888888	888888	888888	888888	888888	888888	7	888888	888888	888888	888888	888888	888888	888888	888888	888888	888888	888888	888888
12884156	IVIG	Placebo	10	11	3	888888	888888	888888	888888	888888	888888	888888	888888	888888	888888	888888	888888	999999	999999	999999	999999	999999	999999	999999	999999	999999	999999	999999	999999	999999
21847649	Hydrocortisone	Placebo	251	248	4	42	43	888888	888888	888888	888888	888888	888888	888888	888888	888888	888888	7	80	97	888888	888888	888888	888888	888888	888888	888888	888888	888888	888888
21106341	Intervention HSD	Control NS	50	57	888888	888888	888888	888888	888888	999999	999999	999999	999999	999999	999999	999999	999999	888888	888888	888888	888888	888888	999999	999999	999999	999999	999999	999999	999999	999999
25776532	EGDT	Usual care	625	626	3	84	97	888888	888888	888888	888888	1	888888	888888	1	888888	888888	999999	999999	999999	999999	999999	999999	999999	999999	999999	999999	999999	999999	999999
24108510	Simvastatin	Placebo	146	138	3	888888	888888	888888	888888	888888	888888	888888	1	1	1	4	3	7	888888	888888	888888	888888	888888	888888	888888	1	1	1	14	9
22738085	HES	Ringer's	398	400	4	67	73	888888	888888	1	1	1	1	1	1	888888	888888	5	67	73	888888	888888	1	1	1	1	1	1	888888	888888
25605469	PiCCO	Control	168	182	4	31	36	888888	888888	888888	888888	888888	888888	888888	888888	888888	888888	7	65	81	888888	888888	2	2	2	888888	888888	888888	888888	888888
26444879	oxygen alone	noninvasive ventilation	183	191	3	888888	888888	888888	888888	888888	888888	888888	1	1	1	888888	888888	999999	999999	999999	999999	999999	999999	999999	999999	999999	999999	999999	999999	999999
25241089	Mw	control	25	25	4	888888	888888	888888	888888	888888	888888	888888	888888	888888	888888	888888	888888	10	888888	888888	888888	888888	2	1	2	888888	888888	888888	888888	888888
16878192	Placebo	Dexamethasone	14	15	4	888888	888888	888888	888888	888888	888888	888888	888888	888888	888888	888888	888888	7	888888	888888	888888	888888	888888	888888	888888	1	2	2	3	5
17174015	Se+	Se-	18	22	3	2	1	888888	888888	888888	888888	888888	888888	888888	888888	888888	888888	7	4	1	888888	888888	888888	888888	888888	888888	888888	888888	888888	888888
14999442	SOC	WW	32	32	1	888888	888888	888888	888888	888888	888888	888888	888888	888888	888888	888888	888888	10	888888	888888	888888	888888	888888	888888	888888	888888	888888	888888	888888	888888
12163783	Placebo	C1-inhibitor	20	20	4	888888	888888	888888	888888	888888	888888	888888	1	1	1	888888	888888	999999	999999	999999	999999	999999	999999	999999	999999	999999	999999	999999	999999	999999
23379498	PHVHF	CVVH	15	15	3	888888	888888	888888	888888	888888	888888	888888	888888	888888	888888	888888	888888	999999	999999	999999	999999	999999	999999	999999	999999	999999	999999	999999	999999	999999
17992396	Placebo	Dexamethasone	15	14	4	888888	888888	888888	888888	888888	888888	888888	888888	888888	888888	888888	888888	7	888888	888888	888888	888888	888888	888888	888888	3	1	2	10	3
23351964	Control	Septimeb	13	16	4	888888	888888	888888	888888	888888	888888	888888	1	1	1	888888	888888	10	888888	888888	888888	888888	888888	888888	888888	2	1	2	888888	888888
99999996	Group A/B	Control	50	25	4	888888	888888	888888	888888	888888	888888	888888	888888	888888	888888	888888	888888	10	888888	888888	888888	888888	888888	888888	888888	888888	888888	888888	888888	888888
21118369	HA Group	Control group	24	20	999999	999999	999999	999999	999999	999999	999999	999999	999999	999999	999999	999999	999999	7	888888	888888	888888	888888	888888	888888	888888	888888	888888	888888	888888	888888
17019548	AVP	NE	13	10	2	888888	888888	888888	888888	888888	888888	888888	1	2	2	3	3	999999	999999	999999	999999	999999	999999	999999	999999	999999	999999	999999	999999	999999
22615589	Control group	IMOD group	10	10	4	888888	888888	888888	888888	888888	888888	888888	888888	888888	888888	888888	888888	10	888888	888888	888888	888888	888888	888888	888888	888888	888888	888888	888888	888888
20080034	HD	VHD	10	11	4	888888	888888	888888	888888	888888	888888	888888	1	1	1	1	2	10	888888	888888	888888	888888	888888	888888	888888	1	1	1	1	2
24699555	Normocaloric	Hypocaloric	54	46	4	888888	888888	888888	888888	888888	888888	888888	888888	888888	888888	888888	888888	7	888888	888888	888888	888888	888888	888888	888888	888888	888888	888888	888888	888888
23511280	LiDCO	Control	10	11	1	888888	888888	888888	888888	888888	888888	888888	888888	888888	888888	888888	888888	999999	999999	999999	999999	999999	999999	999999	999999	999999	999999	999999	999999	999999
12225613	Pentaglobin	Control	21	18	4	888888	888888	888888	888888	1	1	1	888888	888888	888888	888888	888888	8	888888	888888	888888	888888	1	1	1	888888	888888	888888	888888	888888
21347869	Se+	Se-	75	75	3	888888	888888	888888	888888	888888	888888	888888	888888	888888	888888	888888	888888	10	888888	888888	888888	888888	888888	888888	888888	888888	888888	888888	888888	888888
25680826	SoC	SFI	24	21	3	888888	888888	888888	888888	888888	888888	888888	888888	888888	888888	888888	888888	7	888888	888888	888888	888888	888888	888888	888888	888888	888888	888888	888888	888888
25027749	Control	NAC	18	21	4	888888	888888	888888	888888	888888	888888	888888	888888	888888	888888	888888	888888	5	888888	888888	888888	888888	888888	888888	888888	888888	888888	888888	888888	888888
21939972	CLRT	Control	14	13	4	888888	888888	888888	888888	888888	888888	888888	888888	888888	888888	888888	888888	5	888888	888888	888888	888888	888888	888888	888888	888888	888888	888888	888888	888888
24484547	Placebo	low & high dose AscA	8	16	4	888888	888888	888888	888888	1	1	1	888888	888888	888888	888888	888888	999999	999999	999999	999999	999999	999999	999999	999999	999999	999999	999999	999999	999999
18687132	AOX	Placebo	102	98	4	888888	888888	888888	888888	888888	888888	888888	888888	888888	888888	888888	888888	5	888888	888888	888888	888888	888888	1	1	888888	888888	888888	888888	888888
23297395	JBS	TTO	196	195	4	888888	888888	888888	888888	999999	999999	999999	999999	999999	999999	999999	999999	10	888888	888888	888888	888888	999999	999999	999999	999999	999999	999999	999999	999999
10321661	Hydrocortisone	Placebo	20	20	4	888888	888888	888888	888888	888888	888888	888888	888888	888888	888888	888888	888888	10	888888	888888	888888	888888	888888	888888	888888	888888	888888	888888	888888	888888
24740134	AB103	Placebo	30	10	3	888888	888888	888888	888888	888888	888888	888888	888888	888888	888888	888888	888888	7	888888	888888	888888	888888	888888	888888	888888	888888	888888	888888	888888	888888
24632673	rh-APC	saline	33	38	999999	999999	999999	999999	999999	999999	999999	999999	999999	999999	999999	999999	999999	5	888888	888888	888888	888888	888888	888888	888888	888888	1	888888	888888	888888
19531784	Polymyxin B hemoperfusion	Conventional therapy	34	30	3	888888	888888	888888	888888	888888	888888	888888	0	1	1	0	3	999999	999999	999999	999999	999999	999999	999999	999999	999999	999999	999999	999999	999999
20813485	Saline (SS)	HES	11	9	1	888888	888888	888888	888888	888888	888888	888888	888888	888888	888888	888888	888888	999999	999999	999999	999999	999999	999999	999999	999999	999999	999999	999999	999999	999999
24342495	Control	Antithrombin	30	30	4	888888	888888	888888	888888	888888	888888	888888	1	1	1	888888	888888	10	888888	888888	888888	888888	888888	888888	888888	1	1	1	888888	888888
22624531	HES	NaCl	88	86	4	888888	888888	888888	888888	888888	888888	888888	888888	888888	888888	888888	888888	999999	999999	999999	999999	999999	999999	999999	999999	999999	999999	999999	999999	999999
18504551	Treatment group	Control group	36	36	3	888888	888888	888888	888888	888888	888888	888888	888888	888888	888888	888888	888888	9	13	17	888888	888888	2	2	2	888888	888888	888888	888888	888888
17205018	N-acetylcysteine	Placebo	71	71	4	888888	888888	888888	888888	1	1	1	888888	888888	888888	888888	888888	10	888888	888888	888888	888888	1	1	1	888888	888888	888888	888888	888888
23348980	Atorvastatin	Placebo	123	127	3	888888	888888	888888	888888	888888	888888	888888	888888	888888	888888	888888	888888	10	888888	888888	888888	888888	1	888888	1	888888	888888	888888	888888	888888
26325537	Standard medical treatment	High-volume plasmafiltration	90	92	3	888888	888888	888888	888888	888888	888888	888888	1	1	1	888888	888888	7	888888	888888	888888	888888	888888	888888	888888	2	1	1	888888	888888
20705780	AF+	AF-	21	21	4	888888	888888	888888	888888	888888	888888	888888	888888	888888	888888	888888	888888	5	888888	888888	888888	888888	888888	888888	888888	888888	888888	888888	888888	888888
25091790	De-escalation	Continuation	59	57	999999	999999	999999	999999	999999	999999	999999	999999	999999	999999	999999	999999	999999	8	888888	888888	888888	888888	2	2	2	1	1	1	888888	888888
21445641	Selenite	Placebo	15	16	3	888888	888888	888888	888888	888888	888888	888888	888888	888888	888888	888888	888888	10	888888	888888	888888	888888	888888	888888	888888	888888	888888	888888	888888	888888
24912653	FFP	no FFP	38	38	1	888888	888888	888888	888888	888888	888888	888888	888888	888888	888888	888888	888888	999999	999999	999999	999999	999999	999999	999999	999999	999999	999999	999999	999999	999999
26553084	PCT	Control	30	30	2	888888	888888	888888	888888	888888	888888	888888	888888	888888	888888	888888	888888	999999	999999	999999	999999	999999	999999	999999	999999	999999	999999	999999	999999	999999
16276166	Hydrocortisone	Placebo	18	23	4	888888	888888	888888	888888	888888	888888	888888	888888	888888	888888	888888	888888	5	888888	888888	888888	888888	888888	888888	888888	888888	888888	888888	888888	888888
15286541	Adsorber group	Standard group	65	76	4	888888	888888	888888	888888	888888	888888	888888	1	1	1	5	4	7	888888	888888	888888	888888	888888	888888	888888	1	1	1	6	5
19114908	Treatment	Control	45	45	4	888888	888888	888888	888888	888888	888888	888888	1	1	1	888888	888888	9	14	11	888888	888888	1	1	1	1	1	1	4	3
23558909	Control	GDT	26	26	888888	888888	888888	888888	888888	999999	999999	999999	999999	999999	999999	999999	999999	888888	888888	888888	888888	888888	999999	999999	999999	999999	999999	999999	999999	999999
22136422	Probiotic	Control	26	26	4	888888	888888	888888	888888	888888	888888	888888	1	888888	1	1	888888	8	888888	888888	888888	888888	888888	888888	888888	1	1	1	2	1
23095370	CVVH	CVVHD	35	38	4	11	12	888888	888888	888888	888888	888888	888888	888888	888888	888888	888888	7	23	26	888888	888888	888888	888888	888888	888888	888888	888888	888888	888888
23939351	Restrictive	Liberal	51	49	888888	888888	888888	888888	888888	999999	999999	999999	999999	999999	999999	999999	999999	888888	888888	888888	888888	888888	999999	999999	999999	999999	999999	999999	999999	999999
26385664	Xenon	Sevoflurane	21	21	1	888888	888888	888888	888888	888888	888888	888888	888888	888888	888888	888888	888888	999999	999999	999999	999999	999999	999999	999999	999999	999999	999999	999999	999999	999999
26853286	NE	AVP	15	15	2	888888	888888	888888	888888	888888	888888	888888	2	1	2	6	2	999999	999999	999999	999999	999999	999999	999999	999999	999999	999999	999999	999999	999999
12122526	Dopexamine	Control	52	50	4	888888	888888	888888	888888	888888	888888	888888	1	1	1	11	6	7	888888	888888	888888	888888	2	2	2	2	1	2	16	11
19559792	Rosiglitazone	Control	12	9	2	888888	888888	888888	888888	0	0	0	888888	888888	888888	888888	888888	10	888888	888888	888888	888888	0	0	0	888888	888888	888888	888888	888888
22366046	Cooling	No cooling	101	99	888888	888888	888888	888888	888888	888888	888888	888888	888888	888888	888888	888888	888888	888888	888888	888888	888888	888888	888888	888888	888888	888888	888888	888888	888888	888888
23711230	VCV	ASV	25	23	888888	888888	888888	888888	888888	999999	999999	999999	999999	999999	999999	999999	999999	888888	888888	888888	888888	888888	999999	999999	999999	999999	999999	999999	999999	999999
18007263	Experimental (I+R)	Control	27	28	4	888888	888888	888888	888888	888888	888888	888888	888888	888888	888888	888888	888888	10	888888	888888	888888	888888	888888	888888	888888	888888	888888	888888	888888	888888
22692171	Meropenem alone	Meropenem plus moxifloxacin	273	278	4	61	69	888888	888888	888888	888888	888888	1	1	1	888888	888888	10	149	153	888888	888888	2	2	2	1	1	1	888888	888888
26373705	intervention	control	44	44	4	888888	888888	888888	888888	888888	888888	888888	1	1	1	888888	888888	7	888888	888888	888888	888888	888888	888888	888888	1	1	1	888888	888888
16915107	Control	Albumin	50	50	4	888888	888888	888888	888888	888888	888888	888888	888888	888888	888888	888888	888888	7	888888	888888	888888	888888	888888	888888	888888	888888	888888	888888	888888	888888
24781725	Levosimendan	Placebo	17	16	3	888888	888888	888888	888888	3	1	2	888888	888888	888888	888888	888888	999999	999999	999999	999999	999999	999999	999999	999999	999999	999999	999999	999999	999999
10766680	Invasive management	Clinical management	204	209	3	888888	888888	888888	888888	888888	888888	888888	1	1	1	888888	888888	7	888888	888888	888888	888888	888888	888888	888888	1	1	1	888888	888888
21474219	Control	EPA-GLA diet	71	61	3	888888	888888	888888	888888	888888	888888	888888	888888	888888	888888	888888	888888	7	888888	888888	888888	888888	888888	888888	888888	888888	888888	888888	888888	888888
24408035	Control	Fish oil	30	30	3	888888	888888	888888	888888	888888	888888	888888	1	1	1	888888	888888	7	888888	888888	888888	888888	888888	888888	888888	1	1	1	888888	888888
19573904	Etomidate	Ketamine	234	235	3	888888	888888	888888	888888	0	0	0	1	1	1	888888	888888	999999	999999	999999	999999	999999	999999	999999	999999	999999	999999	999999	999999	999999
20663207	APC	Placebo	16	16	999999	999999	999999	999999	999999	999999	999999	999999	999999	999999	999999	999999	999999	5	888888	888888	3	4	1	1	1	1	0	1	888888	888888
26809247	Fixateur externe	Nail	17	16	4	888888	888888	888888	888888	1	1	1	888888	888888	888888	888888	888888	10	888888	888888	888888	888888	1	3	2	888888	888888	888888	888888	888888
21658010	Glutamine	Control	205	208	999999	999999	999999	999999	999999	999999	999999	999999	999999	999999	999999	999999	999999	10	161	164	888888	888888	3	3	3	1	1	1	888888	888888
25959848	Conventional bundle	Improved bundle	52	53	3	888888	888888	888888	888888	888888	888888	888888	888888	888888	888888	888888	888888	999999	999999	999999	999999	999999	999999	999999	999999	999999	999999	999999	999999	999999
25851818	Optimized	control	20	23	3	888888	888888	888888	888888	888888	888888	888888	888888	888888	888888	888888	888888	999999	999999	999999	999999	999999	999999	999999	999999	999999	999999	999999	999999	999999
21926601	Control	Hydrocortisone	49	48	2	10	9	888888	888888	1	1	1	1	1	1	1	2	999999	999999	999999	999999	999999	999999	999999	999999	999999	999999	999999	999999	999999
23327199	Control	Ta1	180	181	3	888888	888888	888888	888888	888888	888888	888888	1	1	1	888888	888888	7	888888	888888	888888	888888	888888	888888	888888	1	1	1	31	16
26015869	study group	control group	23	23	3	888888	888888	888888	888888	888888	888888	888888	0	0	0	0	0	9	888888	888888	5	2	888888	888888	888888	0	0	0	0	0
25080051	Inhaled nitric oxide	Sham	26	23	1	2	1	888888	888888	888888	888888	888888	1	1	1	2	1	999999	999999	999999	999999	999999	999999	999999	999999	999999	999999	999999	999999	999999
25096691	IMHP	HP	152	149	4	13	30	888888	888888	888888	888888	888888	888888	888888	888888	888888	888888	10	72	76	888888	888888	888888	888888	888888	888888	888888	888888	888888	888888
24105455	Sirolimus	Nonsirolomus	19	19	3	0	0	0	0	0	0	0	0	0	0	0	0	7	0	2	888888	888888	888888	888888	888888	888888	888888	888888	888888	888888
25538412	Treatment	Control	7	8	3	888888	888888	888888	888888	888888	888888	888888	888888	888888	888888	888888	888888	999999	999999	999999	999999	999999	999999	999999	999999	999999	999999	999999	999999	999999
25032942	microcirculation targeted resuscitation	standard resuscitation	37	28	4	888888	888888	888888	888888	888888	888888	888888	888888	888888	888888	888888	888888	999999	999999	999999	999999	999999	999999	999999	999999	999999	999999	999999	999999	999999
25561281	Blood purification	Control	98	97	888888	888888	888888	888888	888888	888888	888888	888888	888888	888888	888888	888888	888888	888888	888888	888888	888888	888888	888888	888888	888888	888888	888888	888888	888888	888888

late_day	late_all_g1	late_all_g2	late_comp_g1	late_comp_g2	late_LOS_g1	late_LOS_g2	late_LOS_all	late_death_g1	late_death_g2	late_death_all	late_death_g1_exact	late_death_g2_exact	patients_excluded_from_general_analysis_set	patients_excluded_spec	SOFA-Range	ICU discharge	discrepancy	notes
14	888888	888888	888888	888888	3	3	3	1	1	1	19	14	1	6 (group 1)/5 (group 2), due to withdrawn consent; other missing values due to death excluded from specific analysis (see cat3_excl)	20	0	discrepancy between analyzed patients on day 3 (107 vs. 103) and deceased patients on day 2 (13 vs. 11), we assumed that for some of the deceased patients SOFA value was available on day 3.	t10_all only reported for per-protocol analysis (6 patients in group 1 excluded, though SOFA data could still have been gathered/used for intention-to-treat analysis, hence no exact value available)
999999	999999	999999	999999	999999	999999	999999	999999	999999	999999	999999	999999	999999	0		24	0		SOFAc & SOFA evaluated (20 & 24 point range)
999999	999999	999999	999999	999999	999999	999999	999999	999999	999999	999999	999999	999999	0		20	0		multiple intervention groups
999999	999999	999999	999999	999999	999999	999999	999999	999999	999999	999999	999999	999999	0		24	0		regular SOFA score (0-24) & extended cardiovascular subscore (0-8)
999999	999999	999999	999999	999999	999999	999999	999999	999999	999999	999999	999999	999999	0		24	0		
28	194	186	888888	888888	888888	888888	888888	2	2	2	86	78	0		24	0		missing values differ greatly between subscores
888888	888888	888888	888888	888888	999999	999999	999999	999999	999999	999999	999999	999999	0	3 (1 missing allocation ID, 2 not receiving intervention)	24	1	unclear when/how frequent SOFA was assessed	as ICU discharge=1 and only descriptives (mean & median) were reported, only cat1 missing data could apply (to some degree)
999999	999999	999999	999999	999999	999999	999999	999999	999999	999999	999999	999999	999999	1	randomisation: n(g1)=630, n(g2)=630; excluded: n(g1)=5 (3 requested removal of data, 2 declared ineligible), n(g2)=4 (1 requested removal of data, 3 declared ineligible)	24	0	discrepancy between mentioned missing SOFA values on day 3 (84 vs. 97) and percentage of patients discharged before day 3 (more than 25% in each group). We assumed SOFA values were available for patients discharged before day 3.	
14	888888	888888	888888	888888	888888	888888	888888	1	1	1	22	16	0		24	0	discipancy of handling missing individual SOFA components, we registered as Patients with missing individual SOFA components were excluded	
999999	999999	999999	999999	999999	999999	999999	999999	999999	999999	999999	999999	999999	1	4 excluded after randomisation (no consent given, met exclusion criteria), another 2 excluded after intervention/before analysis (withdrawn consent)	20	0	according to appendix (data) most outcomes w/ full data sets, yet other information (Table S3, appendix) suggests lower patient no. on t4/10 than at baseline, hence missing data likely	appendix (protocol) outlines planned strategies to handle missing SOFA data, presumably these were indeed employed which could explain the discrepancy between lower patient no. and claims of complete outcome data (see "discrepancy")
999999	999999	999999	999999	999999	999999	999999	999999	999999	999999	999999	999999	999999	0		24	0		
999999	999999	999999	999999	999999	999999	999999	999999	999999	999999	999999	999999	999999	0		24	0		
28	888888	888888	888888	888888	4	4	4	2	2	2	7	8	0		24	0		
999999	999999	999999	999999	999999	999999	999999	999999	999999	999999	999999	999999	999999	0		24	0	discipancy of handling missing individual SOFA components, we registered as no method is used	
14	7	7	888888	888888	888888	888888	888888	888888	888888	888888	888888	888888	0		24	0		
999999	999999	999999	999999	999999	999999	999999	999999	999999	999999	999999	999999	999999	0		24	0		
999999	999999	999999	999999	999999	999999	999999	999999	999999	999999	999999	999999	999999	0		20	0	SOFA included all domains, but scored from 4-24 (not 0-24)	
999999	999999	999999	999999	999999	999999	999999	999999	999999	999999	999999	999999	999999	0		24	0		
999999	999999	999999	999999	999999	999999	999999	999999	999999	999999	999999	999999	999999	0		24	0	No measurement taken from patients after ICU discharge, but no clear indication of how/if missing measurements were compensated for	
14	888888	888888	888888	888888	888888	888888	888888	3	1	2	7	3	0		24	0		No clear description of statistics; LOCF not employed due to missing data, but to calculate delta SOFA
28	888888	888888	888888	888888	1	1	1	888888	888888	888888	888888	888888	0		24	0		Intervention groups A & B summed
14	888888	888888	888888	888888	888888	888888	888888	888888	888888	888888	888888	888888	0		24	0		
999999	999999	999999	999999	999999	999999	999999	999999	999999	999999	999999	999999	999999	0		20	0		
14	888888	888888	888888	888888	888888	888888	888888	888888	888888	888888	888888	888888	0		24	0		
999999	999999	999999	999999	999999	999999	999999	999999	999999	999999	999999	999999	999999	1	1 (g2) did not meet enrollment criteria	24	0		
999999	999999	999999	999999	999999	999999	999999	999999	999999	999999	999999	999999	999999	0		24	0		
999999	999999	999999	999999	999999	999999	999999	999999	999999	999999	999999	999999	999999	0		24	0		
999999	999999	999999	999999	999999	999999	999999	999999	999999	999999	999999	999999	999999	1	3 in control due to technical problems	24	0	3 patients excluded according to methods section, yet full control group (n=21) mentioned in results tables?	
28	888888	888888	888888	888888	888888	888888	888888	2	2	2	888888	888888	0		24	0		
999999	999999	999999	999999	999999	999999	999999	999999	999999	999999	999999	999999	999999	0		24	0		
999999	999999	999999	999999	999999	999999	999999	999999	999999	999999	999999	999999	999999	0		24	0		
999999	999999	999999	999999	999999	999999	999999	999999	999999	999999	999999	999999	999999	0		24	0		
999999	999999	999999	999999	999999	999999	999999	999999	999999	999999	999999	999999	999999	1	2 (discontinued intervention)	24	0		Intervention groups high & low dose summed
999999	999999	999999	999999	999999	999999	999999	999999	999999	999999	999999	999999	999999	0		24	0		
28	888888	888888	888888	888888	999999	999999	999999	999999	999999	999999	999999	999999	0		24	1	unclear if daily SOFA assessment until ICU or hospital discharge (yet ICU discharge=1 applies either way); max. stay even for ICU=446 days, for this analyisis tmind11_day was set to max. 28 days as IQR=3/11	since ICU discharge=1 & no specific timepoint analysis was conducted, only cat1 missing data could apply (single missed assessments)
14	888888	888888	888888	888888	888888	888888	888888	1	1	1	888888	888888	0		24	0	repeated measures-statistic mentioned in text, but mixed model (incl. Between-groups) used for comparison	fixed value used systematically to estimate GCS-subscale of SOFA due to inability to measure adequately, but not used to compensate for missed assessments
28	888888	888888	888888	888888	888888	888888	888888	1	1	1	2	2	1	3 (1 Leukocytopenia, 1 received dose higher than planned, 1 did not meet diagnostic criteria)	24	0		intervention groups 1 & 2 combined for this investigation
15	888888	888888	888888	888888	888888	888888	888888	1	1	1	888888	888888	0		24	0		
999999	999999	999999	999999	999999	999999	999999	999999	999999	999999	999999	999999	999999	0		24	0		
999999	999999	999999	999999	999999	999999	999999	999999	999999	999999	999999	999999	999999	1	4 (2 in each group) excluded due to death & 1 (group B) due to differential diagnosis	24	0		
28	888888	888888	888888	888888	888888	888888	888888	1	1	1	888888	888888	0	999999	24	0		
999999	999999	999999	999999	999999	999999	999999	999999	999999	999999	999999	999999	999999	1	12 (group 1)/10 (group 2) excluded, efficacy analysis (incl. SOFA) only included patients reaching primary endpoint (hemodynamic stability)	24	0		
12	13	19	888888	888888	2	2	2	888888	888888	888888	888888	888888	1	18 (9 each group) excluded due to LOS<3days	24	0		
24	888888	888888	888888	888888	1	1	1	888888	888888	888888	888888	888888	0		24	0	discrepancy of last observed SOFA value: last observation of SOFA is described in methods with 20, in figure 1 last one is 24; we considered 24 days.	
14	888888	888888	888888	888888	2	2	2	888888	888888	888888	888888	888888	0		20	0		
999999	999999	999999	999999	999999	999999	999999	999999	999999	999999	999999	999999	999999	0		24	0		2 studies outlined in this article, only study A was assessed (study B = proof-of-principle study not assessing SOFA); CLIF-SOFA score also included in study A, but not assessed in this analysis
999999	999999	999999	999999	999999	999999	999999	999999	999999	999999	999999	999999	999999	0		24	0		
999999	999999	999999	999999	999999	999999	999999	999999	999999	999999	999999	999999	999999	1	1 (group 1)/3 (group 2) due to freedom deprivation	24	0		in this study death during ICU stay was interpreted as discharge on that day (and only non-discharged patients appear to have been included in statistics), for this analysis we assumed both discharge and death to indeed be among the excluded SOFA scores (i.e. cat2_excluded & cat3_excluded)
999999	999999	999999	999999	999999	999999	999999	999999	999999	999999	999999	999999	999999	1	1 (group 1)/3 (group 2) due to errors in enrolment	24	0		
999999	999999	999999	999999	999999	999999	999999	999999	999999	999999	999999	999999	999999	1	2 (group 1)/3 (group 2) excluded due to no intervention	24	0	mortality according to Table 4 (clinical outcomes 24hs after randomization) would be 51 (group 1) & 71 (group 2), yet Table 4 includes other variables clearly not measured at 24hs (e.g. length of stay, mechanical ventilation days), hence t4_death was registered as unknown (888888)	
999999	999999	999999	999999	999999	999999	999999	999999	999999	999999	999999	999999	999999	0		24	0		
999999	999999	999999	999999	999999	999999	999999	999999	999999	999999	999999	999999	999999	1	5 (group 1)/2 (group 2) due to death, lost to follow-up, false inclusion	24	0		
28	888888	888888	888888	888888	1	1	1	2	2	2	19	19	1		24	0	LOCF employed for other outcome variables, but not for missing SOFA scores	2 patients died before assessment, registered as excluded from general analysis, not as cat3_excluded
28	37	35	888888	888888	3	3	3	1	1	1	8	7	0		24	0	not entirely clear whether missing values due to death at various timepoints (Figure 1) are reported as additive or accumulative/total, for this investigation interpreted as accumulative/total numbers	
999999	999999	999999	999999	999999	999999	999999	999999	999999	999999	999999	999999	999999	1	6 (group 1)/6 (group 2), e.g. due to protocol violations & exlcusion criteria	24	1		unclear at which timepoints SOFA was actually measured/only max. values during ICU stay were analysed (ICU discharge=1, thereby also only cat1 missing data could apply) & as mean ICU stay=30/42hs tmind11 likely does not apply at all
21	888888	888888	888888	888888	1	1	1	1	1	1	3	4	0		24	0		very unlikely that ICU discharge means missing data due to LOS in this study, since patients discharged from hospital entirely are explicitely mentioned as missing data
999999	999999	999999	999999	999999	999999	999999	999999	999999	999999	999999	999999	999999	1	4 (group 1)/1 (group 2), e.g. due to early death	24	0		custom SOFA score (modified in various ways)
888888	888888	888888	888888	888888	999999	999999	999999	999999	999999	999999	999999	999999	0		20	1		no fixed day of SOFA measurement, entire stay until discharge/death assessed (ICU discharge=1) & delta baseline-maximum SOFA during stay used for evaluation, hence only cat1 missing data could apply
999999	999999	999999	999999	999999	999999	999999	999999	999999	999999	999999	999999	999999	0		24	0		
999999	999999	999999	999999	999999	999999	999999	999999	999999	999999	999999	999999	999999	0		24	0		
999999	999999	999999	999999	999999	999999	999999	999999	999999	999999	999999	999999	999999	0		24	0	Figure 1 lists n=52/50 for SOFA, though clearly data are missing at t4/t10	unclear whether SOFA scores could still be assessed from non-survivors at t4/t10, due to specific wording (non-/survivors) here deaths on-day are interpreted as missing data (assuming the differentiation survivor/non-survivor at t4 was conducted before assessing SOFA scores)
42	888888	888888	888888	888888	1	3	2	2	3	2	3	5	1	1 (received different intervention)	24	0	Note, GEE would not be appropriate in the presence of missing data unless the analyssi was weighted according to the inverse probabiltiy of missing	
14	888888	888888	888888	888888	888888	888888	888888	1	2	2	888888	888888	0		24	0		delta baseline-maximum SOFA during stay & presumably also fixed baseline-day 14 delta used for evaluation, but no indication for other timepoints
888888	888888	888888	888888	888888	999999	999999	999999	999999	999999	999999	999999	999999	0		24	1		timepoints for assessment & calculation of delta SOFA unclear, possibly ICU discharge=1
888888	888888	888888	888888	888888	888888	888888	888888	888888	888888	888888	888888	888888	0		24	0		
14	184	197	888888	888888	3	3	3	1	1	1	51	52	0		24	1		generally ICU discharge=1 with mean of all values (i.e. likely no missing scores), but multivariate analysis was conducted investigating SOFA at tmind11, hence missing data applies
15	888888	888888	888888	888888	2	2	2	1	1	1	888888	888888	0		24	0	tmind11_day=15 according to paper, but =14 according to supplementary material	SOFA assessed daily, but analysis according to specific timepoints (t10 & tmind11)
28	49	49	888888	888888	3	3	3	2	1	1	15	12	0		24	0		
999999	999999	999999	999999	999999	999999	999999	999999	999999	999999	999999	999999	999999	1	2 (group 1)/2 (group2), e.g. due to emergency operations	24	0	SOFA range described as 4-24 (range=20) in-text, but no description for where points were cut/mentioning in protocol, hence SOFA-range probably =24	
28	888888	888888	888888	888888	888888	888888	888888	2	2	2	63	81	1	5, withdrawn consent	24	0		
21	888888	888888	888888	888888	3	3	3	888888	888888	888888	888888	888888	1	9 (group 1)/19 (group 2), e.g. not receiving intervention/mechanical ventilation & protocol violations	24	0		SOFA measured daily, but only delta at specific days evaluated, hence t4=3/t10=7/tmind11=21
13	888888	888888	888888	888888	1	1	1	1	1	1	888888	888888	0		20	1		SOFA measured daily until discharge/max. 14 days, but ΔSOFA-analysis conducted for specific days, hence missing data could apply & hence t4=3/t10=7/tmind11=13; also no clear description what overall ΔSOFA represents (possibly last-first value, max.-first value, etc.)
999999	999999	999999	999999	999999	999999	999999	999999	999999	999999	999999	999999	999999	1	94 (group 1)/92 (group 2), e.g. LOS<3days/death before reaching hospital	24	0		patients systematically excluded when LOS<3days, hence t4_LOS=0 & patients_excluded=1; but no additional missing values due to LOS, hence cat2_excluded=0
999999	999999	999999	999999	999999	999999	999999	999999	999999	999999	999999	999999	999999	0		24	0	discrepancy of missing data: table 2: comparision of 16 vs 16 for day 10, but at least some patients discharged from hospital before day 10 (minimum of ICU stay is 0 days and minimum hospital stay is 2 days), we decided therefore that missing SOFA due to discharge occurred	hospital stay range 2-140, hence t10_LOS=1 (also t10_death=1), but n=100% at t10 according to paper; missing components at t10 reported & handled through LOCF, which likely also explains complete SOFA scores at t10 despite LOS/death
28	888888	888888	888888	888888	3	3	3	888888	888888	888888	888888	888888	1	1 (group 2), death before intervention	20	0		
999999	999999	999999	999999	999999	999999	999999	999999	999999	999999	999999	999999	999999	1	5 (no data available)	24	0		LOS derived from duration of intervention (supplementation) as described in paper; full SOFA & without GCS were reported, as well as single subscores to minimize impact of missing data, but this analysis focused on total SOFA scores
999999	999999	999999	999999	999999	999999	999999	999999	999999	999999	999999	999999	999999	0		24	0		
999999	999999	999999	999999	999999	999999	999999	999999	999999	999999	999999	999999	999999	1	4 (group 1)/3 (group 2), e.g. due to missing data	24	0		
999999	999999	999999	999999	999999	999999	999999	999999	999999	999999	999999	999999	999999	1	1 (group 1)/1 (group 2), e.g. due to differing interventions	24	0		primary outcome assessed SOFA (sub-)score as IV, specific subscore as additional outcome measure
999999	999999	999999	999999	999999	999999	999999	999999	999999	999999	999999	999999	999999	1	4 (group 1)/2 (group 2), e.g. due to withdrawn consent	24	0		
14	888888	888888	11	9	888888	888888	888888	0	0	0	0	0	0		24	0	"Since all of the patients survived the first 2 weeks, there are no missing outcomes", yet number of cases with missing PaO2/FiO2 ratio reported for t10/tmind11 (SOFA subscore), hence some components are missing & unclear how this was handled	
999999	999999	999999	999999	999999	999999	999999	999999	999999	999999	999999	999999	999999	0		20	0		3 patients died before drug administration, but were included in analysis, hence t4_death>0
999999	999999	999999	999999	999999	999999	999999	999999	999999	999999	999999	999999	999999	0		24	0		
999999	999999	999999	999999	999999	999999	999999	999999	999999	999999	999999	999999	999999	0		24	0		
999999	999999	999999	999999	999999	999999	999999	999999	999999	999999	999999	999999	999999	0		24	0		
999999	999999	999999	999999	999999	999999	999999	999999	999999	999999	999999	999999	999999	1	13 (group 1)/12 (group 2), e.g. due to differential diagnosis & protocol violation	24	0		
888888	888888	888888	888888	888888	888888	888888	888888	888888	888888	888888	888888	888888	0		24	0		no indication exactly when SOFA was assessed, hence also no information regarding missing data mentioned/to be derived

column name	type	code	descritpion
	numeric	888888 = unknown, 999999 = not applicable	
PubMed ID	numeric		PubMed ID
author	text		Name of first author
year_published	numeric		Year published
nr	numeric		order according de Grooth [2]
SOFA derivative_1	numeric	1=fixed SOFA, 2=delta SOFA, 3=mean SOFA, 4=median SOFA, 5=maximum SOFA, 6=sum SOFA, 7=SOFA time course	used SOFA derivative
SOFA derivative_2	numeric	1=fixed SOFA, 2=delta SOFA, 3=mean SOFA, 4=median SOFA, 5=maximum SOFA, 6=sum SOFA, 7=SOFA time course	other used SOFA derivative (if applicable)
SOFA derivative_3	numeric	1=fixed SOFA, 2=delta SOFA, 3=mean SOFA, 4=median SOFA, 5=maximum SOFA, 6=sum SOFA, 7=SOFA time course	other used SOFA derivative (if applicable)
primary endpoint	numeric	1=yes, 0=no	SOFA used as primary endpoint (according to paper)
statistical method_1	numeric	1=Wilcoxon signed-rank test (WSR), 2=Mann-Whitney-U-Test (MWU), 3=mixed models, 4=linear regression, 5=mainly descriptive statistics, 6=Analysis of Covariance (ANCOVA), 7=between-subjects Analysis of Variance (ANOVA), 8=logistic regression, 9=within-subjects t-test, 10=between-subjects t-test, 11=non-parametric analysis of variance (e.g. Kruskal-Wallace), 12=odds ratio, 13=chi-squared, 14=correlation (Spearman's R), 15=within-subjects Analysis of Variance (ANOVA)	statistical method used to analyse SOFA score
statistical method_2	numeric	1=Wilcoxon signed-rank test (WSR), 2=Mann-Whitney-U-Test (MWU), 3=mixed models, 4=linear regression, 5=mainly descriptive statistics, 6=Analysis of Covariance (ANCOVA), 7=between-subjects Analysis of Variance (ANOVA), 8=logistic regression, 9=within-subjects t-test, 10=between-subjects t-test, 11=non-parametric analysis of variance (e.g. Kruskal-Wallace), 12=odds ratio, 13=chi-squared, 14=correlation (Spearman's R), 15=within-subjects Analysis of Variance (ANOVA)	other statistical method used to analyse SOFA score (if applicable)
statistical method_3	numeric	1=Wilcoxon signed-rank test (WSR), 2=Mann-Whitney-U-Test (MWU), 3=mixed models, 4=linear regression, 5=mainly descriptive statistics, 6=Analysis of Covariance (ANCOVA), 7=between-subjects Analysis of Variance (ANOVA), 8=logistic regression, 9=within-subjects t-test, 10=between-subjects t-test, 11=non-parametric analysis of variance (e.g. Kruskal-Wallace), 12=odds ratio, 13=chi-squared, 14=correlation (Spearman's R), 15=within-subjects Analysis of Variance (ANOVA)	other statistical method used to analyse SOFA score (if applicable)
statistical method_compensation	numeric	1=yes, 0=no	statistical method used to compensate for missing data due to single missed assessment, early discharge from ICU or death (e.g. multiple imputation, linear model assessing differences in available data, cumulative incidence curves for death as competing risk prior to resolution)
statistical method_IV	numeric	1=yes, 0=no	SOFA score used as independent variable or covariate in statistical analysis (e.g. multiple logistic regression)?
reas1_mean_adjacent	numeric	1=yes, 0=no	Is mean of adjacent values used to replace missing SOFA values?
reas1_LOCF	numeric	1=yes, 0=no	Is LOCF used to replace missing SOFA values?
reas1_fixed	numeric	1=yes, 0=no	Is a fixed value used to replace missing SOFA values?
reas1_excluded	numeric	1=yes, 0=no	Are missing SOFA components explicitely values?
reas2_fixed	numeric	1=yes, 0=no	Is LOCF used to replace missing SOFA values due to early discharge?
reas2_LOCF	numeric	1=yes, 0=no	Is a fixed value used to replace missing SOFA values due to early discharge?
reas2_excluded	numeric	1=yes, 0=no	Are missing SOFA values due to early discharge explicitely excluded?
reas3_fixed	numeric	1=yes, 0=no	Is LOCF used to replace missing SOFA values due to death?
reas3_LOCF	numeric	1=yes, 0=no	Is a fixed value used to replace missing SOFA values due to death?
reas3_excluded	numeric	1=yes, 0=no	Are missing SOFA values due to death explicitely excluded?
name_g1	text		treatment name according to study
name_g2	text		treatment name according to study
n_g1	numeric		number of patients in group g1
n_g2	numeric		number of patients in group g2
early_day	numeric		day of early SOFA score observation (day 1 to 4)
early_all_g1	numeric		number of overall missing SOFA values of group g1 (for time period: day 1 to 4)
early_all_g2	numeric		number of overall missing SOFA values of group g2 (for time period: day 1 to 4)
early_comp_g1	numeric		number of missing SOFA components for group g1 (for time period: day 1 to 4)
early_comp_g2	numeric		number of missing SOFA components for group g2 (for time period: day 1 to 4)
early_LOS_g1	numeric	0=0%,1=>0-24%,2=25%-49%,3=50%-75%,4=75%-100%	percentage of n_g1 w/ missing SOFA values due to early discharge (for time period: day 1 to 4)
early_LOS_g2	numeric	0=0%,1=>0-24%,2=25%-49%,3=50%-75%,4=75%-100%	percentage of n_g2 w/ missing SOFA values due to early discharge (for time period: day 1 to 4)
early_LOS_all	numeric	0=0%,1=>0-24%,2=25%-49%,3=50%-75%,4=75%-100%	percentage of total n (g1+g2) w/ missing SOFA values due to early discharge (for time period: day 1 to 4)
early_death_g1	numeric	0=0%,1=>0-24%,2=25%-49%,3=50%-75%,4=75%-100%	percentage of n_g1 w/ missing SOFA values due to death (for time period: day 1 to 4)
early_death_g2	numeric	0=0%,1=>0-24%,2=25%-49%,3=50%-75%,4=75%-100%	percentage of n_g2 w/ missing SOFA values due to death (for time period: day 1 to 4)
early_death_all	numeric	0=0%,1=>0-24%,2=25%-49%,3=50%-75%,4=75%-100%	percentage of total n (g1+g2) w/ missing SOFA values due to death (for time period: day 1 to 4)
early_death_g1_exact	numeric		number of missing SOFA values due to death of group g1 (for time period: day 1 to 4)
early_death_g2_exact	numeric		number of missing SOFA values due to death of group g2 (for time period: day 1 to 4)
intermediate_day	numeric		day of middle SOFA score observation (day 5 to 10)
intermediate_all_g1	numeric		number of overall missing SOFA values of group g1 (for time period: day 5 to 10)
intermediate_all_g2	numeric		number of overall missing SOFA values of group g2 (for time period: day 5 to 10)
intermediate_comp_g1	numeric		number of missing SOFA components for group g1 (for time period: day 5 to 10)
intermediate_comp_g2	numeric		number of missing SOFA components for group g2 (for time period: day 5 to 10)
intermediate_LOS_g1	numeric	0=0%,1=>0-24%,2=25%-49%,3=50%-75%,4=75%-100%	percentage of n_g1 w/ missing SOFA values due to early discharge (for time period: day 5 to 10)
intermediate_LOS_g2	numeric	0=0%,1=>0-24%,2=25%-49%,3=50%-75%,4=75%-100%	percentage of n_g2 w/ missing SOFA values due to early discharge (for time period: day 5 to 10)
intermediate_LOS_all	numeric	0=0%,1=>0-24%,2=25%-49%,3=50%-75%,4=75%-100%	percentage of total n (g1+g2) w/ missing SOFA values due to early discharge (for time period: day 5 to 10)
intermediate_death_g1	numeric	0=0%,1=>0-24%,2=25%-49%,3=50%-75%,4=75%-100%	percentage of n_g1 w/ missing SOFA values due to death (for time period: day 5 to 10)
intermediate_death_g2	numeric	0=0%,1=>0-24%,2=25%-49%,3=50%-75%,4=75%-100%	percentage of n_g2 w/ missing SOFA values due to death (for time period: day 5 to 10)
intermediate_death_all	numeric	0=0%,1=>0-24%,2=25%-49%,3=50%-75%,4=75%-100%	percentage of total n (g1+g2) w/ missing SOFA values due to death (for time period: day 5 to 10)
intermediate_death_g1_exact	numeric		number of missing SOFA values due to death of group g1 (for time period: day 5 to 10)
intermediate_death_g2_exact	numeric		number of missing SOFA values due to death of group g2 (for time period: day 5 to 10)
late_day	numeric		day of late SOFA score observation (at least 11)
late_all_g1	numeric		number of overall missing SOFA values of group g1 (for time period: at least 11)
late_all_g2	numeric		number of overall missing SOFA values of group g2 (for time period: at least 11)
late_comp_g1	numeric		number of missing SOFA components for group g1 (for time period: at least 11)
late_comp_g2	numeric		number of missing SOFA components for group g2 (for time period: at least 11)
late_LOS_g1	numeric	0=0%,1=>0-24%,2=25%-49%,3=50%-75%,4=75%-100%	percentage of n_g1 w/ missing SOFA values due to early discharge (for time period: at least 11)
late_LOS_g2	numeric	0=0%,1=>0-24%,2=25%-49%,3=50%-75%,4=75%-100%	percentage of n_g2 w/ missing SOFA values due to early discharge (for time period: at least 11)
late_LOS_all	numeric	0=0%,1=>0-24%,2=25%-49%,3=50%-75%,4=75%-100%	percentage of total n (g1+g2) w/ missing SOFA values due to early discharge (for time period: at least 11)
late_death_g1	numeric	0=0%,1=>0-24%,2=25%-49%,3=50%-75%,4=75%-100%	percentage of n_g1 w/ missing SOFA values due to death (for time period: at least 11)
late_death_g2	numeric	0=0%,1=>0-24%,2=25%-49%,3=50%-75%,4=75%-100%	percentage of n_g2 w/ missing SOFA values due to death (for time period: at least 11)
late_death_all	numeric	0=0%,1=>0-24%,2=25%-49%,3=50%-75%,4=75%-100%	percentage of total n (g1+g2) w/ missing SOFA values due to death (for time period: at least 11)
late_death_g1_exact	numeric		number of missing SOFA values due to death of group g1 (for time period: at least 11)
late_death_g2_exact	numeric		number of missing SOFA values due to death of group g2 (for time period: at least 11)
patients_excluded	numeric	1=yes, 0=no	Are patient excluded from the general analysis set (i.e. randomised, but not analysed)?
patients_excluded_spec	text		Description of patient excluded from the general analysis set
SOFA-Range	text		range
ICU discharge	numeric	1=yes, 0=no	there is no fixed day of SOFA observation, the whole ICU duration (until discharge or death) is considered
discrepancy	text		described discrepancy
notes	text		other notes
